# Editorial: Women in head and neck cancer, volume II: 2022

**DOI:** 10.3389/fonc.2023.1278798

**Published:** 2023-09-12

**Authors:** Panagiota Economopoulou, Ioannis Kotsantis, Amanda Psyrri

**Affiliations:** Section of Medical Oncology, Department of Internal Medicine, Faculty of Medicine, National and Kapodistrian University of Athens, Attikon University Hospital, Athens, Greece

**Keywords:** head and neck cancer, HPV, oropharyngeal cancer, oral cancer, sex disparities, erlotinib, papillary thyroid carcinoma

The Research Topic that accompanies this editorial, encompasses 5 articles that mainly focus on sex disparities in head and neck cancer.

Human PapillomaVirus (HPV) has been recognized as a significant contributing factor in squamous cell cancers of the head and neck (SCCHN), particularly of the oropharynx. HPV-related oropharyngeal carcinoma (OPC) has emerged as a distinct disease entity with different pathogenesis, molecular profile and prognosis. More specifically, patients with HPV-positive disease tend to be affected at a younger age, present with extensive nodal involvement albeit with smaller tumors and are characterized by improved clinical outcomes (1). In the current Research Topic, Preissner et al. report a retrospective analysis of over 140000 cases of head and neck cancers, with a subgroup of 62775 cases of OPC, defined as cancer of the base of the tongue, tonsil and oropharynx. The authors compared the prognosis between cancers of these anatomic locations of men and women with or without HPV infection. They found that female compared to male sex has a greater impact on prognosis of HPV-positive cancers in specific anatomic locations, such as base of the tongue and tonsils. This might be interpreted by a lower consumption of tobacco and alcohol by women, which is an adverse prognostic factor in HPV-positive disease.

In the same context, squamous cell carcinoma of the oral cavity represents a malignancy more frequently encountered in men that has been traditionally etiologically linked to tobacco and alcohol consumption. Indeed, according to Global Cancer Statistics, men present with an age-standardized rate of 6.0, whereas the corresponding number for women is 2.3 (2). Tagliabue et al. sought to evaluate differences in survival and prognosis based on sex/gender in a cohort of patients with oral cancer that were treated in a single center in Italy. The authors collected and analyzed demographic and clinical data including hemoglobin levels and neutrophil to lymphocyte ratio from 576 patients, 230 women and 346 men. Interestingly, men were found to have better clinical outcomes that women in early-stage tumors, and low hemoglobulin rates were an independent prognostic factor for women in early-stage disease. No differences were found in stages III-IV, possibly due to poor survival associated with advanced stage disease.

Current studies on SCCHN mainly focus on immunotherapy and combinatorial approaches. However, there is a lack of significant research on targeted therapies. Since the approval of cetuximab as a radiosensitizer in locally advanced SCCHN (3) and as component of the EXTREME regimen in metastatic disease (4), little progress has been achieved. While current efforts are focusing on developing more potent agents targeting EGFR, Porosnicu et al. designed a window-of-opportunity study evaluating erlotinib, an ‘old’ tyrosine kinase inhibitor targeting the epidermal growth factor receptor (EGFR) as a short course neoadjuvant treatment in resectable SCCHN. Erlotinib was administered at two different doses depending on smoking status; more specifically, current smokers received erlotinib at an increased dose of 300 mg. In addition, patients were subjected to positron emission tomography/computed tomography (PET/CT) scan at baseline, 4-7 days post initiation of erlotinib and before surgery. Interestingly, among 19 patients who participated, 11 were responders. In addition, the percentage change in early and post-treatment PET/CT scan as compared to pre-treatment CT scan was significantly associated with radiologic response on post-treatment CTs. This study highlights the importance of window-of opportunity studies, the need for further research on targeted treatments in SCCHN and the utility of radiomics as a biomarker in response to treatment.

On the other hand, papillary thyroid microcarcinoma (PTMC), defined as a papillary thyroid carcinoma measuring ≤ 10 mm in maximum diameter, is traditionally a tumor with excellent prognosis and indolent behavior. However, several tumor characteristics such as lymph node metastasis (LNM) are considered high-risk and require more aggressive treatment, such as surgery and radioactive iodine therapy. Cao et al. sought to investigate differences in proteomics in PTMC tumor tissues with or without LNM. In addition, differences of protein profiles based on whether tumor tissues were derived from men or women were evaluated. Using a direct data-independent acquisition (DIA) proteomics approach to detect differentially expressed proteins (DEPs), the authors found that in tumors with LNM, DEPs were primarily enriched in categories associated with mitochondrial dysfunction and showed downregulated expression of specific proteins with well-known associations to tumor metastasis. Moreover, the proteomics profiles of men and women with PTMC and LNM were largely distinct. More specifically, compared to female PTMC patients with LNM, the DEPs in the cancerous tissues of male PTMC patients with LNM were mostly enriched in the categories of extracellular matrix (ECM) structural constituent, basement membrane (BM) and collagen-containing ECM. In addition, using bioinformatics to analyze the DEPs between male LNM and female LNM, it was revealed that male LNM show enrichment in the GR signaling, GP6 signaling, CDK5 signaling, VEGF signaling, and mTOR signaling pathway. Finally, upregulation of two DEPs, MMRN2 and NID2 was found in male LNM tissues compared to female LNM tissues and correlated with poor prognosis.

In conclusion, the present Research Topic has included several important articles that add new information to current knowledge regarding differences in biology of tumors and prognosis between men and women with head and neck cancer.

## Author contributions

PE: Conceptualization, Data curation, Resources, Writing – original draft, Writing – review & editing. IK: Resources, Writing – review & editing. AP: Conceptualization, Supervision, Writing – review & editing.

## References

[1] Psyrri A Rampias T Vermorken JB. The current and future impact of human papillomavirus on treatment of squamous cell carcinoma of the head and neck. Ann Oncol (2014) 25(11):2101–15. doi: 10.1093/annonc/mdu265 25057165

[2] Sung H Ferlay J Siegel RL Laversanne M Soerjomataram I Jemal A. Global cancer statistics 2020: GLOBOCAN estimates of incidence and mortality worldwide for 36 cancers in 185 countries. CA: A Cancer J Clin (2021) 71(3):209–49. doi: 10.3322/caac.21660 33538338

[3] Bonner JA Harari PM Giralt J Cohen RB Jones CU Sur RK. Radiotherapy plus cetuximab for locoregionally advanced head and neck cancer: 5-year survival data from a phase 3 randomised trial, and relation between cetuximab-induced rash and survival. Lancet Oncol (2010) 11(1):21–8. doi: 10.1016/S1470-2045(09)70311-0 19897418

[4] Vermorken JB Mesia R Rivera F Remenar E Kawecki A Rottey S. Platinum-based chemotherapy plus cetuximab in head and neck cancer. New Engl J Med (2008) 359(11):1116–27. doi: 10.1056/NEJMoa0802656 18784101

